# Preexisting *TP53* mutation in therapy-related acute myeloid leukemia

**DOI:** 10.1007/s00277-014-2191-0

**Published:** 2014-08-20

**Authors:** Eduard Schulz, Karl Kashofer, Ellen Heitzer, Ketaki N. Mhatre, Michael R. Speicher, Gerald Hoefler, Heinz Sill

**Affiliations:** 1 Division of Hematology, Department of Internal Medicine, Medical University of Graz, Auenbruggerplatz 38D, A-8036 Graz, Austria; 2 Institute of Pathology, Medical University of Graz, Auenbruggerplatz 25/1, A-8036 Graz, Austria; 3 Institute of Human Genetics, Medical University of Graz, Harrachgasse 21/VIII, A-8010 Graz, Austria

**Keywords:** Therapy-related myeloid neoplasms, *TP53*, Leukemogenesis

Dear Editor,

Therapy-related myeloid neoplasms (t-MNs) are a unique clinical entity occurring as late complication of chemotherapy and radiotherapy administered for a primary disease [1]. According to the WHO classification, t-MNs are thought to be due to mutational events in hematopoietic stem and precursor cells (HSPCs) induced by these treatments [2]. However, no consistent biomarker has been identified yet that classifies a particular neoplasm as “therapy-related” [3]. This raises the possibility that other mechanisms may also be operational in their pathogenesis. We and others [4], therefore, hypothesized that mutations contributing to leukemic transformation were preexisting in HSPCs of some of these individuals.

In this study, we selected patients with therapy-related AML (t-AML) following cytotoxic treatment of malignant lymphomas as bone marrow (BM) biopsies are routinely performed during their initial staging procedures. We focused on the *TP53* gene which is frequently mutated in t-AMLs exhibiting a potentially important role in leukemogenesis [5–7]. We identified a somatic heterozygous 64-base pair duplication (Fig. 1a) in a 71 year-old male Caucasian patient who suffered from Hodgkin lymphoma 13 years ago treated by chemotherapy and radiotherapy. To search for potential cooperating mutations, we performed Ion Torrent deep sequencing of recurrently mutated genes in AML [8]. However, no further mutations could be identified (see Supplementary Information for list of genes).

**Fig. 1:**
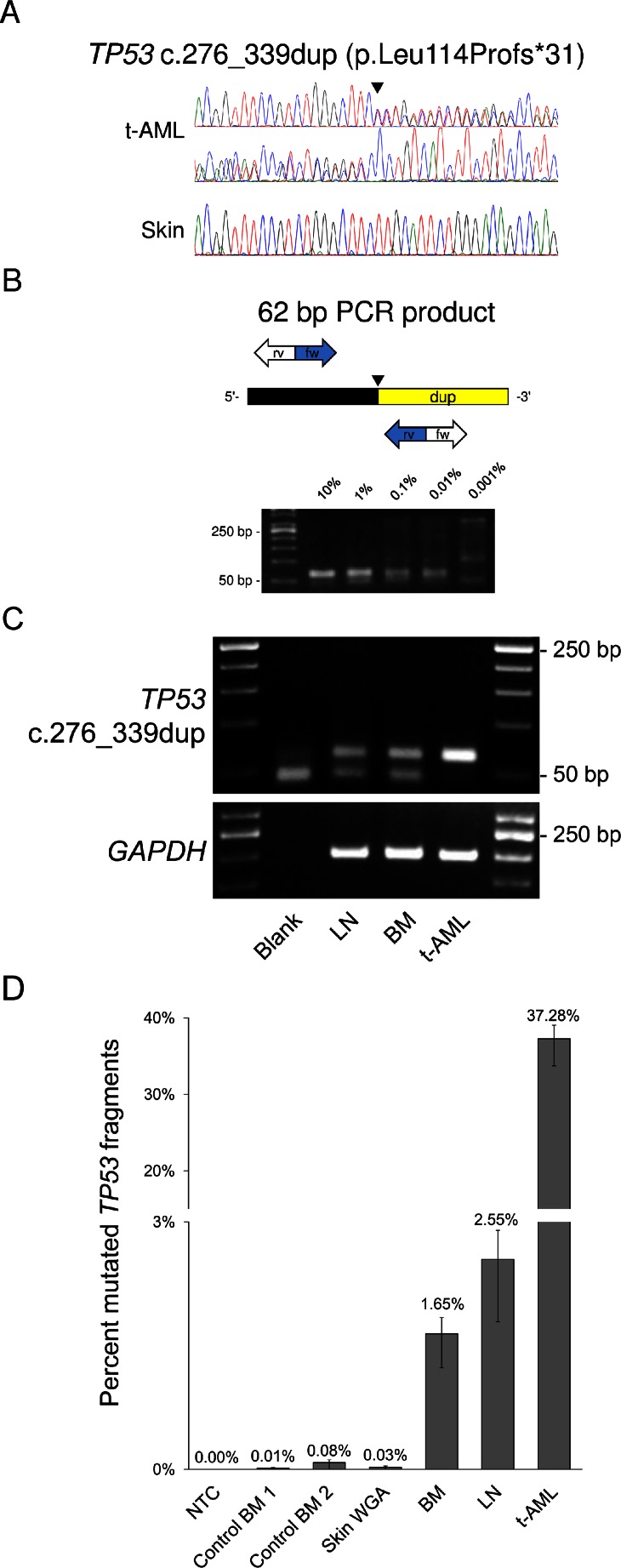
Detection of a preexisting *TP53* mutation in a patient with therapy-related acute myeloid leukemia (t-AML). **a** Bi-directional Sanger sequencing demonstrates the leukemia-specific 64 bp duplication in exon 4 of the *TP53* gene. **b** The primer pair enables the detection of the duplication to a dilution of 0.01 % t-AML DNA in normal control DNA. Analysis of four AML control samples revealed the absence of this duplication in all of them (data not shown). **c** The 62-base pair product (upper band) specific for the *TP53* duplication could be detected in the bone marrow (BM) obtained at diagnosis of Hodgkin lymphoma as well as in a lymphadenitis specimen (LN) the latter obtained 20 years before diagnosis of t-AML. The lower band refers to primer dimers, *GAPDH* (lower gel) served as control gene for DNA integrity. **d** Expansion of cells with the *TP53* duplication determined by digital PCR. Relative ratios between mutant *TP53* fragments and *BRAF* wild-type fragments were assessed using the QuantStudio 3D Digital PCR System (Life Technologies, Carlsbad, CA). Copy number changes at the *BRAF* locus of the t-AML were excluded using low coverage whole genome sequencing to ensure that *BRAF* is suitable as a reference and not subjected to copy number changes. Data shown are means of assays performed in duplicates and error bars indicate 95 % confidence intervals. *BM* bone marrow; *LN* lymphadenitis sample; *NTC* no template control; *WGA* whole genome amplified DNA

We established a highly sensitive PCR assay specific for this rearrangement (Fig. 1b) and could unambiguously demonstrate the presence of the *TP53* mutation in the patient’s BM obtained at the time of the lymphoma staging (Fig. 1c). Surprisingly, the *TP53* duplication was also detected in a reactive lymphadenitis sample obtained 7 years before lymphoma diagnosis (Fig. 1c). To further demonstrate that expansion of the *TP53* mutated clone occurred following cytotoxic treatment, we quantified the *TP53* duplication by digital PCR (dPCR) which showed that the relative proportion of mutated cells increased substantially in the t-AML specimen (Fig. 1d). It, furthermore, confirmed the *TP53* duplication being a somatically acquired event as it was absent from a skin biopsy obtained at the time of the leukemia diagnosis.

In the case report presented here, we were able to demonstrate that cytotoxic treatment did not induce a leukemia-specific mutation but rather may have facilitated the expansion of a pre-leukemic clone harboring a somatic *TP53* mutation. Since dPCR data quantifying the *TP53* duplication were comparable in lymph node and pretreatment BM, the mutation might have occurred in HSPCs that retained their lymphoid as well as myeloid differentiation potential and remained dormant for many years. This finding challenges current concepts of therapy-related leukemogenesis and is in line with data presented at the 2013 annual meeting of the American Society of Hematology [4]. There, somatic *TP53* variants could be identified at low frequencies in mobilized peripheral blood leukocytes of two t-MDS/t-AML cases years before diagnosis. However, in clinical practice, HSPC harvests from peripheral blood are performed following intense chemotherapy including application of recombinant granulocyte-colony factor. Here, we provided definitive evidence that a leukemia-specific mutation could be found in HSPCs before any cytotoxic treatment was administered.

## Electronic supplementary material

Below is the link to the electronic supplementary material.

ESM 1 (PDF 283 kb)
