# Supplementary material for: Preexisting TP53 mutation in therapy-related acute myeloid leukemia
Source: Ann Hematol. 2014 Aug 20;94(3):527–9. doi: 10.1007/s00277-014-2191-0 (PMC4317506; doi:10.1007/s00277-014-2191-0)
Supplement: Supplementary file 1 — (PDF 283 kb) [file 277_2014_2191_MOESM1_ESM.pdf]

Supplementary Information

Annals of Hematology

Pre-existing *TP53* mutation in therapy-related acute myeloid leukemia

Eduard Schulz, Karl Kashofer, Ellen Heitzer, Ketaki N. Mhatre, Michael R. Speicher, Gerald Hoefler, and Heinz Sill

Corresponding author
Prof. Heinz Sill, M.D.

Division of Hematology, Department of Internal Medicine
Medical University of Graz,
Auenbruggerplatz 38D, A-8036 Graz, Austria

T + 43 316 385 80257
F + 43 316 385 14087
E heinz.sill@medunigraz.at

Screening of *TP53* in t-AMLs

The study was approved by the ethics committee of the Medical University of Graz, Austria, and written informed consent was obtained from all patients. We analyzed the *TP53* gene (NM_000546.4) in 15 t-AML specimens that developed post lymphoma treatment by direct sequencing of paired samples using previously published primers and PCR conditions [1]. We identified seven somatically acquired mutations in six patients (40%, 6/15): Six mutations were heterozygous point mutations or a homozygous one nucleotide duplication, respectively (c.455C>T; c.659A>G; c.711G>A; c.818G>A and c.857A>G; c.681_681dupT). However, analyzing bone marrow specimens of the respective patients at the time of lymphoma diagnosis by Ion Torrent deep sequencing (see below) did not result in an unambiguous identification of the particular *TP53* mutations (data not shown). The seventh mutation was the heterozygous 64-base pair duplication (c.276_339dup, p.L114Pfs*56) described in the Letter to the Editor. It was identified in a 71-year-old male Caucasian patient who suffered from Hodgkin lymphoma, clinical stage IA, thirteen years ago. At that time, he was treated with two cycles of COPP/ABVD followed by involved field radiation with a total dose of 30 Grays achieving a complete remission. His secondary malignancy presented with myelodysplasia and transformed to t-AML within six weeks. It exhibited a myelo-monocytic phenotype and the karyotype 46~50,XY,del(5)(q12q33),?r(7)(p22q11)[cp20].

Detection of the *TP53* duplication by PCR

We used standard PCR conditions for the detection of the *TP53* duplication (c.276_339dup, p.L114Pfs*56). DNA concentration was measured using the Quant-iT PicoGreen dsDNA Assay Kit (Life Technologies) and 10 ng were used per reaction. Primers specific for the duplication generating a 62 bp product were designed with Primer3 and as follows: forward, 5'-AAACCTACCAGGGCAGCTAC-3'; reverse, 5’-TGGGAAGGGACAGAAGATGA-3’. To rule out fragmentation of DNA up to 200 bp, we

assessed the DNA integrity of each sample by amplification of a 200 bp product of *GAPDH* as previously described [2].

Ion Torrent targeted deep sequencing

20 ng DNA each of pre-leukemic bone marrow and t-AML with *TP53* duplication were analyzed by targeted deep sequencing. Coding sequences of *ASXL1*, *CEBPA*, *DNMT3A*, *EZH2*, *FLT3*, *HRAS*, *IDH1*, *IDH2*, *JAK2*, *KIT*, *KRAS*, *MLL*, *MPL*, *NPM1*, *NRAS*, *PHF6*, *PTEN*, *RUNX1*, *TET2*, *TP53*, *WT1* were amplified by multiplexed PCR using the AMLv1 Community Panel (Life Technologies). Library preparations were performed using the Ion AmpliSeq Library Kit 2.0 (Thermo Fisher Scientific). Emulsion PCR and sequencing were performed with the appropriate kits (Ion One Touch Template Kit v2 and Ion Proton 200 Sequencing Kit, [both from Thermo Fisher Scientific], respectively) on an Ion Torrent Proton sequencer using a single P1 semiconductor chip yielding reads ranging from 90 to 130 bp consistent with the expected PCR fragment size-range. On average, one million reads were obtained for each sample with more than 90% of bases above AQ20 and 87% to 93% reads on-target. Sequence information was obtained from tumor samples in duplicates and additionally from normal non-tumor material.

Initial data analysis was performed using the Ion Torrent Suite Software (Thermo Fisher Scientific, open source, GPL, https://github.com/iontorrent/). Briefly, this included base calling, alignment to the reference genome (hg19) using the TMAP mapper and variant calling with a modified diBayes approach taking into account the flow space information. All called variants were annotated using open source software (ANNOVAR, http://www.openbioinformatics.org/annovar/; SnpEff, http://snpeff.sourceforge.net/) and custom Perl scripts [3,4].

Quantitative digital PCR

To compare the amount of DNA fragments harbouring the *TP53* duplication between different tissue samples, we performed digital PCR (dPCR) on the QuantStudio

3D platform (Life Technologies) which enables accurate quantitation of a target DNA molecule through partitioning a sample into 20k reactions on a single chip. A TaqMan assay specific for the detection of the *TP53* duplication was designed using Primer Express software version 3.0 (Life Technologies). Primers spanning the breakpoint (TP53_Dup_F, 5'-AGAAAACCTACCAGGGCAGCTAC-3'; TP53_Dup_R, 5'-TCTGGGAAGGGACAGAAGATGA-3') - therefore specific for the detection of the duplication - were synthesized by Microsynth (Balgach, Switzerland). A TaqMan probe (5'-TCCCTGTCATCTTCTGTC-3') located exactly across the breakpoint was designed and synthesized with minor groove binder (MGB) modification and the reporter fluorophore FAM (Life Technologies). To normalize the amount of input DNA, we included a *BRAF* wild type assay (BRAF_WT_F, 5'-CATGAAGACCTCACAGTAAAAATAGGTGAT-3'; BRAF_WT_R, 5'-GGATCCAGACAACTGTTCAAACTGA-3'; BRAF-Probe, 5'-VIC-CCATCGAGATTTCACTGTAG_MGBNFQ-3') on the same chip. Specificity of the assays was tested on a StepOne Plus instrument (Life Technologies) using the TaqMan Genotyping Master Mix (Life Technologies) according to the manufacturer's recommendations. As AMLs harbour frequently copy number changes across the genome, we established a genome-wide copy number profile using low-coverage whole genome sequencing of the t-AML sample in order to assure that the *BRAF* gene was present in two copies [5].

For the dPCR 18 µl of Digital PCR Master Mix (2X) were mixed with 0.45 µl of each primer pair (10 µM), 0.5 µl each of the 6-FAM labelled *TP53* and the VIC labelled *BRAF* MGB TaqMan probes (10 µM), and a total amount of input DNA of 120 ng to a final volume of 36 µl. DNA concentrations were determined using Qubit dsDNA BR Assay Kits (Life Technologies). All samples were run in duplicates, therefore 18 µl each were loaded on two Digital PCR 20k Chip using the QuantStudio 3D Digital PCR Chip Loader (Life Technologies). The chips were then thermal cycled in a two-step PCR using the GeneAmp PCR System 9700, starting with the activation of the polymerase for 10 min at 96 °C, followed by 44 cycles of 56 °C for 2 min and 94 °C for 30 s with a final extension of

2 min at 60 °C. Chips were then imaged in the QuantStudio 3D instrument. Raw data were analyzed using the relative quantification module of the QuantStudio 3D Analysis Suite Software. The confidence level was set to 95% and the desired precision value was 10%.

We used a skin biopsy obtained at the time of t-MN diagnosis as constitutional DNA, however, due to small amounts of DNA, we performed whole genome amplification before dPCR using GenomePlex Complete Whole Genome Amplification (WGA) Kit 2 (Sigma-Aldrich) according to the manufacturers´ recommendations with the following exceptions: we used 5 µl of the DNA sample for library preparation and amplification was performed for 20 cycles. Amplified DNA was purified using Gene Elute PCR Clean-Up Kit (Sigma-Aldrich) and quantified using Qubit dsDNA BR Assay Kits (Life Technologies).

References

1. Schulz E, Valentin A, Ulz P, Beham-Schmid C, Lind K, Rupp V, Lackner H, Wolfler A, Zebisch A, Olipitz W, Geigl J, Berghold A, Speicher MR, Sill H (2012) Germline mutations in the DNA damage response genes BRCA1, BRCA2, BARD1 and TP53 in patients with therapy related myeloid neoplasms. J Med Genet 49(7):422-428. doi:10.1136/jmedgenet-2011-100674

2. van Beers EH, Joosse SA, Ligtenberg MJ, Fles R, Hogervorst FB, Verhoef S, Nederlof PM (2006) A multiplex PCR predictor for aCGH success of FFPE samples. Br J Cancer 94(2):333-337. doi:10.1038/sj.bjc.6602889

3. Wang K, Li M, Hakonarson H (2010) ANNOVAR: functional annotation of genetic variants from high-throughput sequencing data. Nucleic Acids Res 38(16):e164. doi:10.1093/nar/gkq603

4. Cingolani P, Platts A, Wang le L, Coon M, Nguyen T, Wang L, Land SJ, Lu X, Ruden DM (2012) A program for annotating and predicting the effects of single nucleotide

polymorphisms, SnpEff: SNPs in the genome of Drosophila melanogaster strain w1118; iso-2; iso-3. Fly (Austin) 6(2):80-92. doi:10.4161/fly.19695

5. Heitzer E, Ulz P, Belic J, Gutschi S, Quehenberger F, Fischereder K, Benezeder T, Auer M, Pischler C, Mannweiler S, Pichler M, Eisner F, Haeusler M, Riethdorf S, Pantel K, Samonigg H, Hoefler G, Augustin H, Geigl JB, Speicher MR (2013) Tumor-associated copy number changes in the circulation of patients with prostate cancer identified through whole-genome sequencing. Genome Med 5(4):30. doi:10.1186/gm434
